# Regulation of lung inflammation by adiponectin

**DOI:** 10.3389/fimmu.2023.1244586

**Published:** 2023-09-01

**Authors:** Joo-Yeon Lim, Steven P. Templeton

**Affiliations:** Department of Microbiology and Immunology, Indiana University School of Medicine-Terre Haute, Terre Haute, IN, United States

**Keywords:** adiponectin, lung immune response, adiponectin receptors, COPD, asthma, inflammatory pulmonary disease, infectious lung disease, obesity

## Abstract

Adiponectin is an insulin sensitizing hormone that also plays a role in the regulation of inflammation. Although adiponectin can exert pro-inflammatory effects, more studies have reported anti-inflammatory effects, even in non-adipose tissues such as the lung. Obesity is considered an inflammatory disease, is a risk factor for lung diseases, and is associated with decreased levels of plasma adiponectin. The results of recent studies have suggested that adiponectin exerts anti-inflammatory activity in chronic obstructive pulmonary disease, asthma and invasive fungal infection. The signaling receptors of adiponectin, AdipoR1 and AdipoR2, are expressed by epithelial cells, endothelial cells, and immune cells in the lung. In this mini-review, we discuss the anti-inflammatory mechanisms of adiponectin in lung cells and tissues.

## Introduction

Adiponectin (APN) is a protein abundantly produced by adipocytes. It is present at high concentrations in plasma and regulates glucose levels, lipid metabolism, and insulin sensitivity. Plasma APN levels are relatively high in lean and healthy individuals. Decreased circulating levels of APN in obese individuals could enhance the risk of insulin resistance, type 2 diabetes, and cardiovascular disease (1). APN has also been reported to be produced by lymphocytes, skeletal muscle cells, cardiomyocytes, osteoblasts, and liver cells (2–5).

APN binds to signaling adiponectin receptors AdipoR1 and AdipoR2 (6) and the non-signaling receptor T-cadherin (7). While AdipoR1 binds to the globular form of APN, AdipoR2 preferentially binds to the high-molecular weight (HMW) form (8). The HMW isoform is consist of several linked hexamers and trimers (9). Interestingly, females have higher circulating levels of total APN with higher proportions of HMW isoform compares to males (10–12). Unlike the expression of APN, there are no sex differences in the expression of AdipoRs in adipose tissue (13).

Obesity is associated with a higher incidence of various diseases, including pulmonary disorders, such as asthma, chronic obstructive pulmonary disease, and pulmonary hypertension, and is also associated with decreased plasma adiponectin (14–18). Obesity-associated dysregulation of immune responses, inflammatory vigor, and adipose tissue immune cell infiltration are also believed to contribute to infectious disease pathogenesis (19).

According to the World Health Organization (WHO), the two most common chronic lung diseases are COPD and asthma, causing restricted airflow and breathing problem. APN has been implicated to play a role in the pathophysiology of COPD. Many studies revealed decreased serum APN in COPD patients (20). With a steadily increasing population of immunocompromised patients, disease caused by fungal infections remain a great threat to public health. Among lung fungal pathogens, such as *Aspergillus*, *Cryptococcus*, and *Pneumocystis*, Aspergillosis (approximately 57%) by Aspergillus spp. was most common (21). While the APN roles have been more thoroughly investigated in COPD and asthma, fewer studies have implicated in APN lung diseases. APN is required for metabolism and has a regulatory role in inflammation, primarily exerting anti-inflammatory effects. Here, we specifically review published studies that examine the anti-inflammatory activity of APN in inflammatory pulmonary diseases and cells of the lung.

## APN and lung disorder

### APN and COPD

Chronic Obstructive Pulmonary Disease (COPD) is a globally increasing cause of mortality with increasing prevalence over the past 20 years (22). Population aging, smoking and exposure to air pollution are leading risk factors for COPD (22–24). COPD patients showed increased numbers of lung inflammatory cells such as neutrophils, macrophages, and CD8^+^ T cells, with increased production of chemokines and cytokines (25). A mouse model of COPD with tobacco smoke exposure showed increased APN production in bronchoalveolar lavage fluid (BALF) and APN gene expression by airway epithelial cells (26), suggesting that APN has the potential to modulate the inflammatory response in COPD. Another study used an elastase-induced emphysema model to identify the possible role of APN in the pathogenesis of COPD (27). Elastase-induced emphysema is associated with reduced APN concentration. APN-deficient (APN KO) mice show a progressive COPD-like phenotype characterized by progressive emphysema, increased endothelial apoptosis and increased TNF-α activity. APN is associated with inflammation in COPD and is positively correlated with as the neutrophil-recruiting chemokine IL-8 (26). APN can therefore be a biomarker for disease severity and progression in patients of COPD (28).

### APN and asthma

Asthma is a chronic inflammatory disorder that affects airways in the lungs narrowed and swollen by inflammation and blocked by excess mucus. The most common asthma triggers include air pollen, dust products, animal dander, tobacco smoke and a wide range of fungi (29). In an obesity-related asthma mouse model used by Zhu et al., APN level in serum and BALF as well as adiponectin receptor (AdipoR) mRNA expression in lung were decreased and exogenous APN treatment increased both the APN level and AdipoR expression (30). In a mouse model of asthma by Medoff et al., ovalbumin (OVA)-sensitized and challenged APN KO mice exhibited increased pulmonary vascular remodeling, eosinophilic inflammation, and inflammatory chemokine gene expression compared to control mice (31). Furthermore, Obesity is associated with low-grade inflammation and enhances chronic inflammation in the airways of asthmatic obese patients. Expression of AdipoR2 and T-cadherin genes in bronchial epithelial cells was higher among obese patients with asthma than obese controls (32). Obesity in mice was associated with increased BALF macrophages, neutrophils and eosinophils, and increased Th2 cytokine production, including IL-13 and IL-5. Administration of exogenous APN reduced inflammation in obese mice, suggesting a therapeutic potential for the adiponectin pathway (33), and an APN receptor agonist reduced IL-4, IL-17, IL-23, and TNF-α in an OVA/lipopolysaccharide (LPS)-induced obese asthmatic model (34). In summary, APN treatment alleviated pathological changes in lung with reduction of eosinophils, total cell numbers in BALF, the eosinophil-attracting chemokine eotaxin and myeloperoxidase levels, which suggests that APN regulates cell migration into the airway and clearance of pulmonary inflammation in obesity-related asthma (30, 31).

In obese asthmatics, low APN was found more frequently than high (35) and non-obese individuals had higher levels of serum APN compared to obese (36). However, two other studies found no correlation between APN and obesity (37, 38). The differences in results of these studies might be related to demographic differences in age, gender, race, and disease severity. Although clinical data depend mainly on the serum APN, results from animal models and cell cultures demonstrate a potential benefit of APN pathway-stimulating therapy in asthmatics (35).

### APN and pulmonary aspergillosis

In invasive aspergillosis patients, excessive inflammation is associated with increased mortality (39). However, the effects of APN on anti-fungal immune responses in the lung remains unclear.

Fungal spores are easily aerosolized and inhaled (40). Different manifestations of *Aspergillus* infection include allergic bronchopulmonary aspergillosis, chronic pulmonary aspergillosis, and invasive aspergillosis. Allergic bronchopulmonary aspergillosis is considered poorly controlled asthma, while invasive aspergillosis occurs in immunocompromised patients such as those with COPD, solid organ or bone marrow transplant recipients, intensive care unit patients, and patients with severe viral infection (HIV, influenza A virus, COVID-19) (41–45). Invasive aspergillosis has an extremely high mortality rate (50-90%) in immunocompromised patients (46). *Aspergillus fumigatus* is most prevalent and the major cause of aspergillosis (47). APN KO mice with invasive aspergillosis exhibit increased disease pathology including decreased survival rate, increased fungal burden in the lung, increased cytokine production (IL-6 and TNF-α), and increased eosinophil recruitment (48). Aspiration of *Aspergillus fumigatus* conidia or chitin, one of the fungal cell wall components induced increased eosinophil recruitment in APN KO mice compared to wild-type controls (48, 49), and recombinant murine APN inhibited chitin-mediated eosinophil recruitment (49). Thus, it is likely that APN inhibits excessive inflammation in invasive aspergillosis, either directly or indirectly by enhancing antifungal immunity.

**Figure 1:**
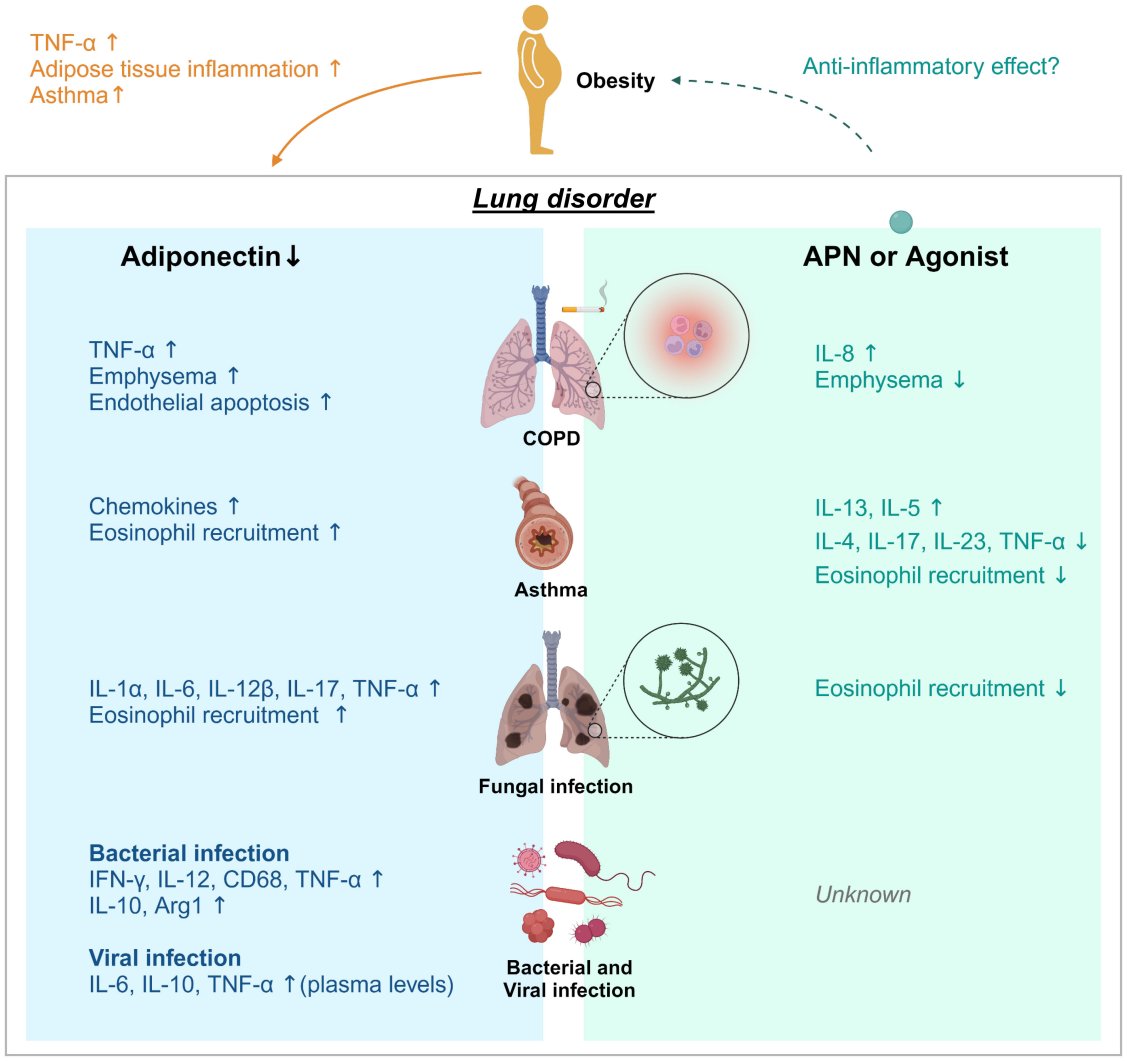
Adiponectin effect on lung disease. Obesity and aging are associated with loss of muscle mass, insulin resistance, and features of metabolic syndromes. Adiponectin (APN) activity is linked to metabolism and inhibition of inflammation. Decreases/deficiencies in plasma APN, even due to obesity, may contribute to adipose tissue inflammation, induction of asthma and TNF-α production in adipose and non-adipose tissues such as the lung. APN regulates inflammation in chronic pulmonary disease (COPD), asthma, fungal, bacterial, and viral infection. Adiponectin deficiency in COPD, aspergillosis, viral infection or tuberculosis resulted in increased expression of the proinflammatory cytokines TNF-α and IL-6, further suggesting that APN functions exerts anti-inflammatory activity. However, the effects of APN or AdipoRon on viral infection remain unknown. Treatment with APN or its agonist affects cytokine production; a decrease of IL-13 and IL-5 in asthma model and an increase of IL-8 in COPD, and increased IL-4, 1L-17, IL-23, and TNF-α in asthma. APN treatment also inhibited lung eosinophil recruitment in response to chitin.

The roles of adiponectin in COPD, asthma, and pulmonary aspergillosis is summarized in **Figure 1**. Recent studies suggest a role for APN in regulating the inflammatory response in aspergillosis, COPD and asthma in either animal models or human patients (28, 35, 38). However, there are limited studies examining the role of APN during fungal infection in murine lungs (48, 49). Moreover, the number of the immunocompromised individuals is rapidly increasing, due to increased use of immunosuppressive therapies. Despite immune suppression, aspergillosis patients can succumb to an uncontrolled inflammatory response. Moreover, COPD and asthma patients showed increased hypersensitivity to *Aspergillus fumigatus* (29, 50, 51). More research is need to further unravel the anti-inflammatory mechanisms of the APN pathway in lung disease, especially in the context of fungal infection.

### APN and bacterial and viral infections

Tuberculosis is a bacterial infectious disease that most often affects the lungs, which is caused by *Mycobacterium tuberculosis*. One-third of the world population is infected with this pathogen. Infection of *M. tuberculosis* alters adipose tissue morphology and contributes to an acute loss of body fat, which worsens pulmonary pathology. Comparing the lungs of *M. tuberculosis-*infected fat-ablated mice to infected fat-unablated mice, the levels of cytokines, such as TNF-α, IFN-γ, CD68, IL-12, and IL-10 were increased (52). APN may promote a reduction of TNF-α in the lungs in *M. tuberculosis*-infected mice (52). In acute lung injury model, APN attenuates LPS-induced lung injury in acute lung injury model (53). These reports suggest that APN regulates pulmonary pathology during bacterial infection.

Epidemiological evidence suggests an association between obesity and increased susceptibility to viral pneumonias associated with influenza, SARS-CoV-2, and COVID-19 cases (19, 54, 55). APN level is reduced in the patients infected with influenza A virus and COVID-19 with respiratory failure (56–58). Overexpression of IL-6, a key adipocyte-secreted inflammatory mediator, is an important risk factor worsening outcomes in influenza virus infection (59). In COVID-19, APN is generated by lymphocytes and downregulates the bone marrow production of granulocytes, with the activation of regulatory T cells (60). While the levels of cytokines in lung in COVID-19 patients have not been analyzed, the plasma levels of IL-6, TNF-α, and IL-10 were highly increased in severe COVID-19 patients with comparable levels to non-COVID-19 patients (61). So far, no study evaluated the effect of APN or AdipoRon on SARS-CoV-2 infection (62). These studies suggest that the APN pathway could be manipulated to provide protection against detrimental inflammation in response to viral infection.

## Anti-inflammatory activity of adiponectin on lung cells

### APN/AdipoRs in lung immune cells

APN and AdipoRs are expressed in the lung, suggesting an important role for the APN pathway in lung biological processes (63). Alveolar macrophages from APN KO mice released higher matrix metalloproteinases and TNF-α, which was suppressed by APN pretreatment (63). In humans, APN release from lung explants is negatively correlated with body mass index (64). Lung macrophages express both of the signaling APN receptors AdipoR1 and AdipoR2 (64). Lung macrophages treated with APN or its AdipoR1/R2 agonist AdipoRon resulted in decreased LPS- and poly (I:C)-induced production of TNF-α, IL-6, CXCL1 and CXCL8 (64). In the murine macrophage-like cell line RAW264, the globular domain of APN binds to the AdipoR1 and inhibits the TLR-induced NF-κB activity (65).

In lean mice, lung regulatory T cells (CD3^+^ CD4^+^ Foxp3^+^ cells) increased AdipoR1 expression, while obese mice with allergic inflammation had reduced AdipoR1 expression in lung regulatory T cells and increased expression in BALF eosinophils (66). Thus, adiponectin exerts anti-inflammatory effects on lung cells, likely due to signaling through adiponectin receptors.

### APN/AdipoRs in lung fibroblast, epithelial cells, and endothelial cells

Idiopathic pulmonary fibrosis is a common pulmonary disease, with high mortality, especially in older people. The idiopathic pulmonary fibrosis mouse model of bleomycin challenge induced a remarkable collagen fiber accumulation with extensive alveolar damage. Furthermore, APN treatment attenuated bleomycin-induced histopathology and inhibits TGF-β1-induced fibrosis in human lung fibroblasts with decreased TNF-α, IL-6, IL-1β, and IL-18 expression (67). Paraquat is an herbicide used worldwide and exposure to paraquat may cause acute injury and fibrosis in humans (68). In a pulmonary fibrosis model induced by paraquat, APN attenuated the fibrosis with decreased TGF-β1 and α-smooth muscle actin by regulating the NF-κB pathway (69). Human lung fibroblasts also express both AdipoR1 and AdipoR2, and APN treatment increased fibroblast expression of AdipoR1 but not AdipoR2. Knockdown of AdipoR1 using siRNA reversed the APN-mediated protective effect against paraquat-mediated fibrosis, demonstrating the importance of the APN-AdipoR1 pathway in fibroblasts for protection against pulmonary fibrosis (69).

In contrast, airway epithelial cells express significant levels of APN and AdipoR1, but not AdipoR2 in COPD patients and cultures of human lung epithelial cells (26). APN attenuated A549 cellular apoptosis and ameliorated cytotoxic effects induced by TNF-α and IL-1β by inhibiting NF-κB transactivation through AdipoR1. APN increased mRNA expression of the anti-inflammatory cytokine IL-10 in lung epithelial A549 cells (70). In summary, these studies provide evidence for a direct effect of APN on proliferation and inflammation of A549 epithelial cells with a protective role of APN in lung cells.

Hallmark features of acute lung injury include immune and endothelial cell activation and loss of vascular integrity (71). When acute lung injury was induced in control and APN KO mice by administration of LPS, APN KO mice appeared more ill with increased BALF protein concentration, increased production of TNF-α and IL-6, and decreased level of IL-10 in lung homogenates (53). Compared to the control mice with LPS injection, increased expression of IL-6, E-selectin, and Nox2 were detected in endothelial cells of APN KO mice (33). APN localizes to pulmonary vascular endothelium and APN deficiency leads to an age-dependent inflammatory vascular phenotype (72). APN deficiency also impairs mitochondrial function, promotes endothelial cell activation, and increases the susceptibility to LPS-induced acute lung injury (73). These finding suggests that parenchymal cells of the lung may play a role in the protective actions of APN.

### Molecular mechanism of APN action on lung disease

In obesity-related asthma mice, APN treatment relieved inflammation and improved AMPK activity with a decrease in iNOS, Bcl-2, and NF-κB levels in lung (30). Treatment of Compound C, the AMPK inhibitor, reverses the effects of APN (30). It has been reported that APN directly binds to AdipoR1 and mediates signaling by activating AMPK.

*M. tuberculosis* infection increases the levels of both PPAR-γ and PPAR-α, key regulators of adipogenesis and lipid oxidation in the lungs (52). In LPS-induced acute lung injury model, NF-κB activation is APN is increased in APN KO mice (53).

In healthy mice, APN treatment induces the activation of p38 MAPK in Helios^−^ regulatory T cells and upregulates the expression of AdipoR1 (74). In human epithelial cell line A549, activation of the APN-AdipoR1 pathway reduces cytotoxic effects inhibiting NF-κB activation and cytokine gene expression through ERK1/2 and AKT (70).

## Conclusions and perspectives

There is increasing data suggesting that APN exerts an anti-inflammatory effect in the lung. Lung cells, including immune cells, epithelial cells, and endothelial cells, express AdipoRs, indicating they also have signaling ability upon binding to APN or an AdipoR agonist such as AdipoRon. Although both pro- and anti-inflammatory properties have been reported, anti-inflammatory function of APN was mainly observed in lung cells (**Table 1**). APN can trigger the activation of AMPK, PPAR, ERK, and AKT through its receptors. It is well known that APN improves different lung diseases; enhancing the signaling might prove a therapeutic target. However, further research will clarify the roles and mechanism of APN pathway-induced protection in lung diseases, including fungal, bacterial, and viral infection, which could result in novel therapies that protect against infection, excessive inflammation, and other lung pathologies.

**Table 1:** Effects of APN in different lung cell types.

Cell types	Cell source	Function	Reference
**Alveolar macrophages**	APN KO mice	APN suppresses high matrix metalloproteinases and TNF-α	(63)
**Human lung macrophages**	LPS- and poly (I:C) infection *in vitro*	APN inhibits infection-induced production of TNF-α, IL-6, CXCL1 and CXCL8	(64)
**Murine macrophage-like cells RAW264**	Cell line	APN binds to AdipoR1 and inhibits the TLR-induced NF-κB activity	(65)
**Lung regulatory T cells**	Obese mice challenged with ovalbumin	Allergic inflammation reduces AdipoR1 expression	(66)
**Human lung fibroblasts HELF**	TGF-β1-induced fibrosis *in vitro*	APN inhibits the production of TNF-α, IL-6, IL-1β and IL-18	(67)
**Human lung fibroblasts WI-38**	Paraquat-induced fibrosis *in vitro*	APN increase AdipoR1 but not AdipoR2	(69)
**Airway epithelial cells A549**	Cell line	APN induces release of IL-8, and IL-10	(26)
		APN attenuates apoptosis and inhibits TNF-α and IL-1β by inhibiting NF-κB activity through AdipoR1	(70)
**Endothelial cells**	APN KO mice with LPS injection	Increased production of IL-6, E-selectin, and Nox2 is detected	(53)

APN, Adiponectin; AdipoR, adiponectin receptors; APN KO mice, Adiponectin-deficient mice; LPS, Lipopolysaccharide.

## Author contributions

J-YL and ST wrote the manuscript. J-YL prepared illustrations. J-YL and ST revised the manuscript. All authors contributed to the article and approved the submitted version.
